# Debate: Social media in children and young people – time for a ban? Beyond the ban – empowering parents and schools to keep adolescents safe on social media

**DOI:** 10.1111/camh.70032

**Published:** 2025-09-10

**Authors:** Katrina E. Champion, Louise Birrell, Scarlett Smout, Maree Teesson, Tim Slade

**Affiliations:** ^1^ The Matilda Centre for Research in Mental Health and Substance Use University of Sydney Sydney NSW Australia; ^2^ Sydney School of Public Health University of Sydney Sydney NSW Australia

**Keywords:** Social media, adolescents, parents, digital literacy

## Abstract

In this article, we examine Australia's landmark decision to ban social media access for children under the age of 16, set to take effect in December 2025. While the legislation aims to protect young people from the harms of social media, including its impact on mental health and wellbeing, the evidence base underpinning the ban remains inconclusive, with most studies unable to establish causality. Drawing on parallels with adolescent alcohol prevention, we argue that prohibition alone is unlikely to be effective. Instead, we advocate for a harm minimisation approach that equips young people with digital literacy, resilience, and help‐seeking skills. We highlight the essential roles of parents, schools, and adolescents in fostering safer social media use and call for inclusive, co‐designed education initiatives.

## Introduction

In a global first, in 2024 Australia passed legislation to ban children under the age of 16 from accessing social media platforms, including Facebook, Instagram, Snapchat, TikTok, X and YouTube. Technology companies will face fines of up to $50 million if they fail to take reasonable steps to prevent children under 16 from creating accounts. The legislation was introduced, in part, in response to growing concerns about the negative impact of social media on young people's mental health and wellbeing.

While the government's intent is clear, the evidence base underpinning the decision is inconclusive (Valkenburg, Meier, & Beyens, 2022). While some research links adolescent social media use with mental health problems, systematic reviews and meta‐analyses show that overall, these associations are weak and inconsistent across studies (Valkenburg et al., 2022). Crucially, it remains unclear whether social media *causes* poor mental health in youth, or whether the association is bi‐directional or influenced by other factors (Thimm‐Kaiser & Keyes, 2025). To date, most studies have used cross‐sectional designs and focused narrowly on how much time young people spend online, rather than how they use social media platforms (Maheux et al., 2025). As a result, we still do not fully understand whether social media directly contributes to mental health issues in young people. More robust longitudinal research is urgently needed to unpack these complex relationships.

## Will the ban work to protect youth mental health?

Regardless of the current state of evidence, the legislation will come into effect in December 2025. This raises a critical question: will the ban be effective? The short answer is we do not know. While the legislation aims to protect young people from the potential harms of social media, its effectiveness remains uncertain.

Possible benefits include shielding youth from online risks that may affect their health and wellbeing, including social comparison and body image concerns, cyberbullying, advertising of harmful products, and exposure to sexualised and/or misogynistic content (Osborne, 2025). The ban may also offer broader benefits, such as reclaiming time for other positive activities (e.g. sleep, physical activity and face‐to‐face interactions with peers and family). The ban has been widely supported by parents, many of whom find it difficult to manage their children's social media use. In our own research, parents commonly reported technology use, especially social media, as the biggest challenge facing adolescents today (Champion et al., 2023). These lived experiences, while not always captured in the academic literature, have contributed to growing public concern.

However, the ban is not without limitations. Adolescents today are ‘digital natives’, and will likely find ways to circumvent the policy, and the proposed age verification systems are not foolproof. There is also concern that the ban covers some, but not all social media platforms, and adolescents will be pushed into less well‐regulated areas where exposure to harmful content could be equally, if not more, concerning. Emerging research also points to the potential positive role that social media can play in promoting good mental health in young people, including destigmatising mental health, and providing access to resources (Hamilton et al., 2024). Importantly, the legislation does not equip young people with the skills to navigate online spaces safely and responsibly, which are essential in today's digital landscape.

## Learning from alcohol prevention initiatives

While the social media ban may help some teenagers and families, it is unlikely to be a panacea. We propose a broader, harm minimisation approach involving parents, schools, and young people. Indeed, the ban's momentum offers a chance to drive broader conversations about responsible technology use. While there are important differences, we may be able to draw lessons and parallels from existing bans imposed on adolescents, such as those related to alcohol use. Decades of alcohol prevention efforts offer valuable insights for addressing digital safety in adolescents. Historically, prevention programs, particularly in the United States, relied on abstinence‐only approaches, promoting ‘zero tolerance’ and ‘just say no’ messaging (Beck, 1998). These strategies have consistently failed to produce meaningful behavioural change. In contrast, harm minimisation approaches acknowledge the realities of adolescent behaviour and aim to equip young people with the tools to navigate alcohol exposure safely. While abstinence remains a desirable goal, this approach accepts that some young people will experiment with alcohol before the legal drinking age. Such prevention efforts focus on minimising harm by providing accurate information, practical skills, and strategies for staying safe and seeking help (Teesson et al., 2017).

Key learnings from this model could be applied to social media use. Just as some adolescents perceive alcohol use as socially beneficial, many view social media as a vital tool for connection and self‐expression (Hamilton et al., 2024). Instead of relying solely on restrictions, we advocate for a harm minimisation approach that promotes digital literacy, resilience, and help‐seeking among young people. This approach recognises that some underage engagement with social media is inevitable and seeks to reduce associated risks through education and skill‐building. The legislation presents a timely opportunity to equip 13‐ to 15‐year‐olds with these skills, just before they gain access at age 16, ensuring they are prepared to navigate social media safely and responsibly. These efforts must be complemented by stronger regulation and greater accountability from technology providers to ensure that digital environments are safe, just as alcohol producers must meet safety standards in advertising and distribution.

## The role of parents, schools and adolescents

While the legislation rightly places greater accountability on social media providers, parents must remain central to efforts to reduce digital harms. Empowering parents and caregivers to better understand social media and the potential benefits and risks is essential. Education efforts should be inclusive and accessible, recognising the diverse needs and digital literacy levels of families.

Supporting parents to have meaningful conversations with their children is also critical. Open, ongoing conversations between parents and adolescents provide an opportunity to co‐develop expectations around technology, screen time and social media use. Rather than relying on surveillance or restriction alone, fostering trust through conversation can empower young people to make safer, more informed choices online. It is possible that the ban may unintentionally discourage disclosure about negative online experiences if adolescents circumvent the rules and fear consequences. Maintaining open lines of communication, even when rules are broken, will be essential to ensuring young people feel able to seek help when needed.

Parents' own use of social media and technology use can impact teenager's behaviours (Arundell, Parker, Timperio, Salmon, & Veitch, 2020). When parents limit and are mindful of their own screen use, discuss this openly with their child, and engage in alternative activities, they model healthy habits that help teenagers learn to use technology in more balanced and constructive ways. Not all young people have access to a supportive home environment, so schools also serve as a critical setting for promoting digital literacy. By providing safe spaces for discussion and skill‐building, schools can complement parental efforts and reinforce positive behaviours. Finally, an essential component of any response to adolescent social media use is the meaningful inclusion of adolescents themselves in design and implementation. Co‐design has been shown to improve the success of adolescent‐targeted initiatives through ensuring that messaging is relevant, accessible, and salient (Guo et al., 2024).

## Conclusion

While the verdict on Australia's social media ban remains uncertain, prohibition alone is rarely a complete solution. Regardless of its impact, education remains essential to help young people and their families navigate digital spaces safely and responsibly. The ban has brought welcome attention to the complexities of adolescent social media use, creating an opportunity to advance conversations around digital safety and wellbeing. To build on this momentum, we call for greater investment in youth and parent education and teacher training. A coordinated, harm minimisation approach, grounded in evidence and inclusive of adolescents, parents, schools, and technology providers, is needed to create safer, healthier digital environments for all young Australians.

## Conflict of interest statement

MT is Co‐Director of Climate Schools Pty Ltd and OurFutures Institute Ltd.

## Funding information

KEC is funded by a University of Sydney Horizon Fellowship. LB and MT are supported by Investigator Grants from the Australian National Health and Medical Research Council (grants 2016301 and 1195284). SS is supported by a philanthropic grant from the BHP Foundation for the Mentally Healthy Futures Project.

## Ethics statement

No ethical approval was required for this debate article.

## Data Availability

Data sharing is not applicable to this article as no new data were created or analysed.
